# PPARγ regulates the expression of genes involved in the DNA damage response in an inflamed endometrium

**DOI:** 10.1038/s41598-022-07986-8

**Published:** 2022-03-07

**Authors:** Karol Mierzejewski, Łukasz Paukszto, Aleksandra Kurzyńska, Zuzanna Kunicka, Jan P. Jastrzębski, Karol G. Makowczenko, Monika Golubska, Iwona Bogacka

**Affiliations:** 1 Department of Animal Anatomy and Physiology, Faculty of Biology and Biotechnology, University of Warmia and Mazury in Olsztyn, Oczapowskiego 1a, 10-719 Olsztyn, Poland; 2 Department of Plant Physiology, Genetics and Biotechnology, Faculty of Biology and Biotechnology, University of Warmia and Mazury in Olsztyn, Oczapowskiego 1a, 10-719 Olsztyn, Poland

**Keywords:** Inflammation, Gene expression

## Abstract

Inflammation is a biological response of the immune system, which can be triggered by many factors, including pathogens. These factors may induce acute or chronic inflammation in various organs, including the reproductive system, leading to tissue damage or disease. In this study, the RNA-Seq technique was used to determine the in vitro effects of peroxisome proliferator-activated receptor gamma (PPARγ) ligands on the expression of genes and long non-coding RNA, and alternative splicing events (ASEs) in LPS-induced inflammation of the porcine endometrium during the follicular phase of the estrous cycle. Endometrial slices were incubated in the presence of LPS and PPARγ agonists (PGJ_2_ or pioglitazone) and a PPARγ antagonist (T0070907). We identified 169, 200, 599 and 557 differentially expressed genes after LPS, PGJ_2_, pioglitazone or T0070907 treatment, respectively. Moreover, changes in differentially expressed long non-coding RNA and differential alternative splicing events were described after the treatments. The study revealed that PPARγ ligands influence the LPS-triggered expression of genes controlling the DNA damage response (*GADD45β, CDK1, CCNA1, CCNG1, ATM*). Pioglitazone treatment exerted a considerable effect on the expression of genes regulating the DNA damage response.

## Introduction

Inflammation is a biological reaction to disrupted tissue homeostasis, caused by interactions between numerous factors^1^. According to the World Health Organization (WHO), chronic inflammatory diseases affecting any organ of the body constitute the greatest health problem and one of the leading causes of death in the world^2^. Inflammation of the female reproductive organs occurs physiologically during the menstrual/estrous cycle, but acute or chronic inflammations and bacterial infections contribute to infertility, endometrial polyps, miscarriage and diseases related to abnormal placentation^1^. Research has demonstrated that bacterial toxicity can be triggered by lipopolysaccharide (LPS), an endotoxin present in the outer membrane of Gram-negative bacteria^3^. Lipopolysaccharide induces the expression of genes regulating the production of cytokines, adhesion proteins and enzymes involved in pro-inflammatory responses^4^. This endotoxin also triggers the release of reactive oxygen species (ROS), reactive nitrogen species (RNS) and nitric oxide (NO)^5^.

Inflammation is self-limiting and usually subsides after harmful particles have been removed and tissue repair is complete. However, prolonged homeostatic imbalance in tissues can ultimately lead to chronic inflammation, increased macrophage recruitment, accelerated aging, apoptosis, unregulated growth and tissue repair. Large quantities of persistent ROS produced by inflammatory cells can damage host cell macromolecules (DNA as well as RNA, lipids, carbohydrates and proteins)^6^. To avoid the above deleterious events, cells have evolved various DNA repair and DNA damage response (DDR) pathways to maintain genomic integrity. The following mechanisms are usually triggered in response to oxidative DNA damage: (1) base excision repair (BER)/single-strand break repair (SSBR), (2) nucleotide excision repair (NER), (3) mismatch repair (MMR), (4) homologous recombination (HR), and (5) nonhomologous end joining (NHEJ)^7^. There is evidence to indicate that LPS leads to DNA damage in mouse embryos and uterine cells during the preimplantation stage, resulting in poor embryonic development and inadequate uterine horn preparation^8^.

Inflammation-related abnormalities in the reproductive system have been frequently described in various species^9,10^. In women, inflammations are manifested mostly by endometriosis, which is a chronic inflammatory disease^10^. It has been hypothesized that the pathogenesis of endometriosis involves bacterial contamination and LPS from *Escherichia coli* (*E. coli)*^11^. The continuous release of LPS from *E. coli* may increase the concentration of LPS in menstrual blood and endotoxin levels in peritoneal fluid. The LPS/TLR4 complex induces pelvic inflammation and promotes the growth and progression of endometriosis through intracellular adaptor molecules and nuclear factor kappa B (NF-κB) light chain enhancers^11^. Moreover, oxidative stress might promote the progression of endometriosis through the activation of TLRs and the NF-κB signaling pathway^11^. It has also been reported that due to high oxidative stress, endometriotic lesions are more sensitive to DNA damage, which contributes to the accumulation of somatic mutations in various genes. In women with endometriosis, greater genomic damage was observed in eutopic endometrial cells^12^. Research has also shown that DDR and DNA repair pathways are impaired in ectopic lesions and eutopic endometrial cells^13^.

Peroxisome proliferator-activated receptors (PPARs) belong to a family of nuclear receptors that act as transcription factors regulating various biological processes such as inflammation and reproduction^14^. Three isoforms of PPAR—α, β/δ and γ—have been described to date^15^. According to research, PPARγ inactivation in mice leads to embryonic death due to abnormal placental angiogenesis and severe developmental damage^16,17^. In addition, the knockout of PPARγ compromises trophoblasts differentiation and placental maze development^18^. The role of PPARγ in the inflammatory response has attracted considerable attention in recent years^19,20^. Generally, PPARγ ligands inhibit NF-κB activity during the inflammatory process^20^. They also stimulate the expression of various antioxidant enzymes and decrease ROS concentrations^21^. PPARγ ligands have also been reported to affect pro/anti-inflammatory cytokines in the reproductive system.

Our recent studies have shown that PPARγ is engaged in the synthesis of inflammatory mediators in the porcine endometrium under physiological conditions (during the luteal phase of the estrous cycle and early pregnancy)^19^. We have also demonstrated that PPARγ is involved in LPS-stimulated inflammation of the porcine endometrium during the mid-luteal phase of the estrous cycle^22^. PPARγ blocking by a specific antagonist exerted pro-inflammatory effects, and this mechanism involved various intracellular pathways, including the cellular response to IL-1, cell migration and granulocyte chemotaxis. The aim of the present study was to investigate the in vitro effect of PPARγ ligands (natural or synthetic agonists and an antagonist) on the transcriptome profile of the porcine endometrium during LPS-induced inflammation in the follicular phase of the estrous cycle (days 18–20). The effect of PPARγ ligands on alternative splicing events (ASEs) and long non-coding RNA (lncRNA) expression was also analyzed.

## Results

### RNA sequencing data

**Figure 1:**
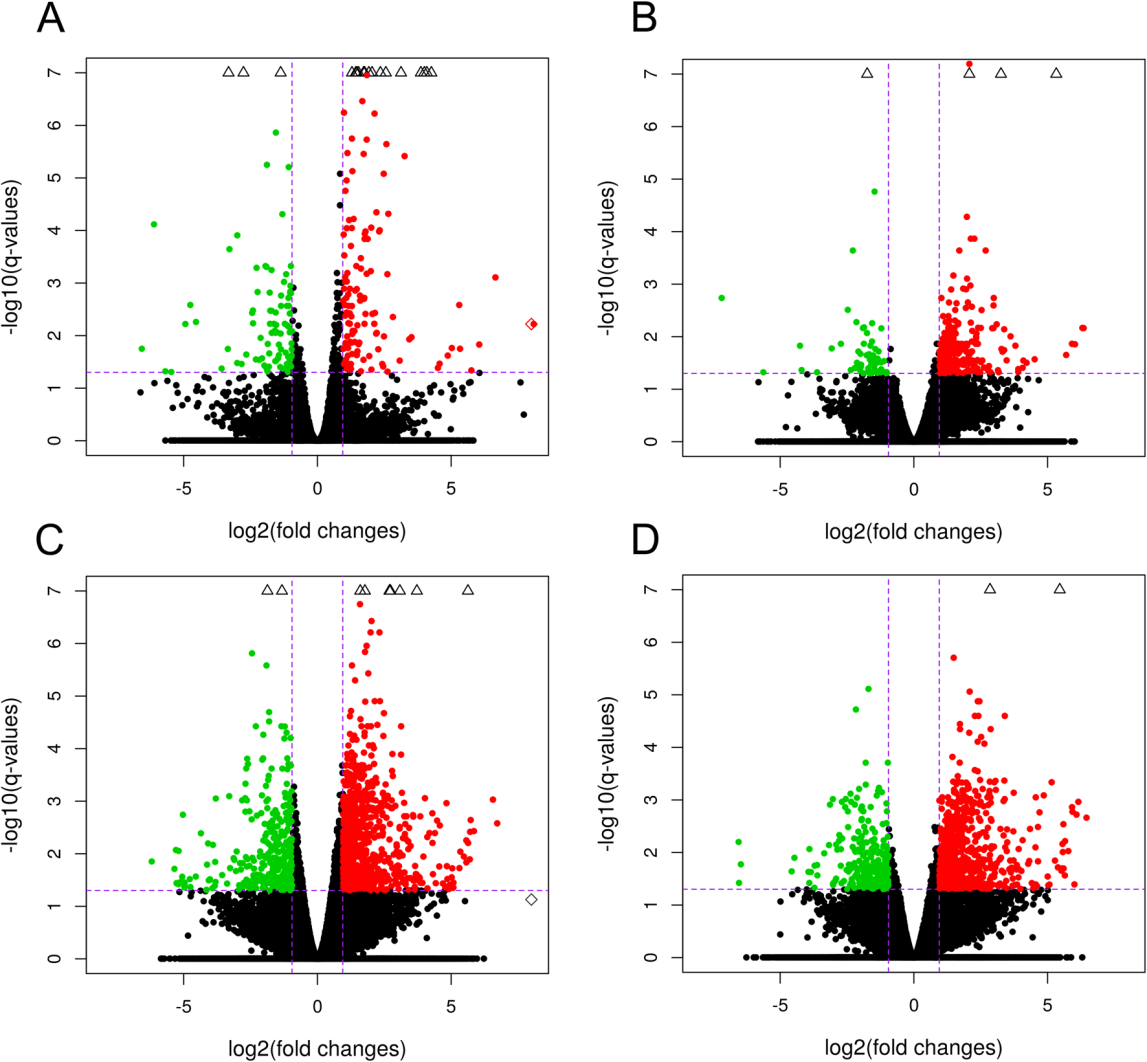
Volcano plots describing the abundance of transcript expression profiles after LPS treatment (**A**); the impact of PPARγ agonists—PGJ_2 _(**B**) or pioglitazone (**C**) and antagonist (**D**) on LPS action in endometrial tissue. Logarithmic fold changes in expression (log_2_FC) are plotted on the X-axis against normalized adjusted *p*-values (Y-axis). The sharp horizontal line denotes the negative logarithmic adjusted *p*-value (0.05) cut-off. Sharp vertical lines denote the fold change cut-off (absolute value of log_2_FC > 1). Points represent gene expression values, where green (underexpressed) and red (overexpressed) points denote significant genes (adjusted *p*-value < 0.05). Grey dots indicate non-significant genes. The plots were generated in base and ggplot2 R bioconductor software.

**Figure 2:**
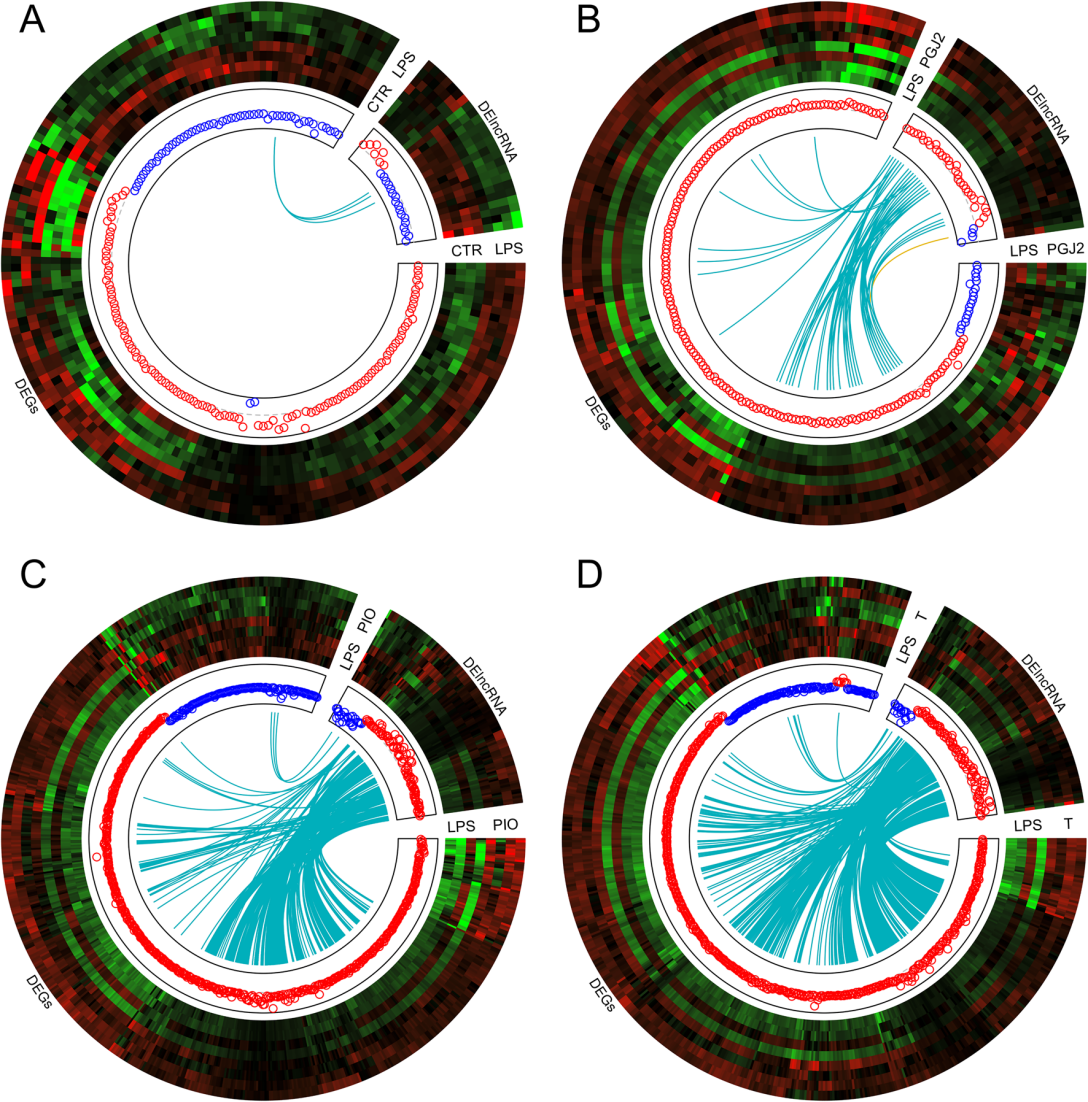
Circular heatmaps presenting the associations between differentially expressed genes (DEGs) and differentially expressed long non-coding RNAs (DElncRNAs) in endometrial tissues treated with LPS (**A**), PPARγ agonists—PGJ_2_ (**B**) or pioglitazone (**C**) and antagonist (**D**). Each circle consists of eight upper tracks presenting the normalized (Z-score; red-green scale) expression profiles for DEGs and DElncRNAs in two separate blocks. The middle tracks describe the expression of upregulated (red) and downregulated (blue) genes in the each compared group. The innermost track presents the correlations between the co-expressed DEGs and DElncRNAs, where blue links depict positive correlations (> 0.9) and orange links depict negative Euclidean correlations (< − 0.9). The plots were generated in circlize R bioconductor software.

**Figure 3:**
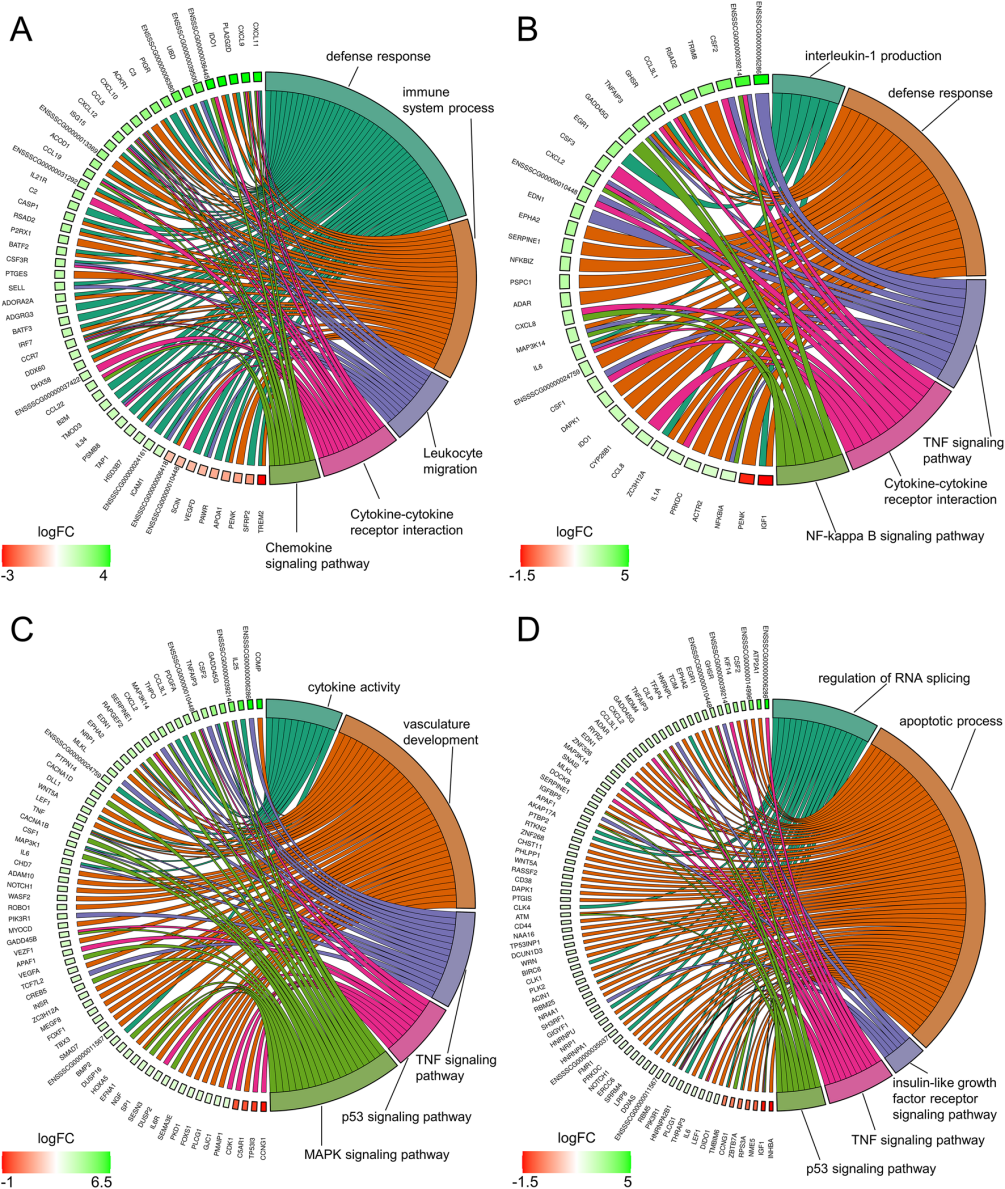
Circos plots presenting selected Gene Ontology processes and KEGG pathways associated with differentially expressed genes (DEGs) engaged in the responses to LPS (**A**), PPARγ agonists PGJ_2_ (**B**) or pioglitazone (**C**) and antagonist (**D**) in endometrial tissue. Logarithmic values (blue-red scale) represent the fold change (logFC) of DEGs. Five-color links combine gene symbols (left) with the most important GO and KEGG annotations (right). The plots were generated in the GOplot R bioconductor software.

RNA sequencing data were prepared for 20 cDNA libraries. They comprised control samples (n = 4; untreated), LPS (n = 4), 15d-prostaglandin J_2_ (n = 4; natural PPARγ agonist; PGJ_2_), pioglitazone (n = 4; synthetic PPARγ agonist; PIO) and T0070907 (n = 4; PPARγ antagonist; T). The sequencing analysis generated 1 114 235 534 raw paired-end reads, with an average of 55.71 million read pairs per sample. The filtered reads were mapped to the Ss11.1.98 version of the porcine genome with an average mapping rate of 93.06%. The analysis revealed that nearly 53.64% of the read pairs were mapped to coding sequences, 6.58% were mapped to introns, 24.67% were mapped to untranslated regions, and the remaining 15.11% were mapped to intergenic regions. Sequencing data and the process of mapping the reads to a reference genome are presented in Tables S1 and S2 in the Supplemental Materials. Volcano plots illustrate changes in gene expression in the LPS-treated group versus the control (untreated) group, as well as in the groups treated with PPARγ ligands (PGJ_2_ or PIO or T) versus LPS (Fig. 1). Circular heatmaps present differentially expressed genes (DEGs) (Fig. 2). Differences in the percentage of splicing inclusion (ΔPSI) values in the above comparisons are presented in the volcano plots of ASEs (Fig. 3).

### The effect of LPS treatment on differential gene expression in the endometrium

**Figure 4:**
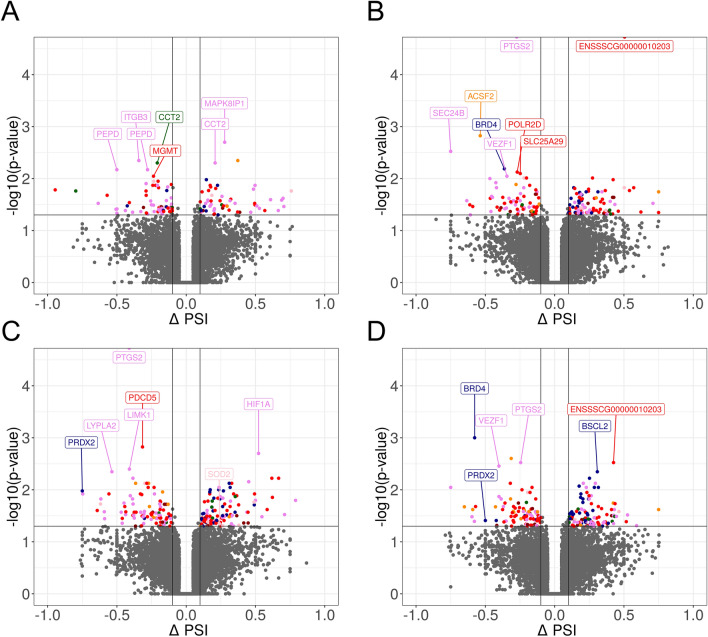
Volcano plot presenting the abundance of alternative splicing events (ASEs) in each experimental comparison: (**A**) LPS versus controls; (**B**) PGJ_2_ versus LPS, (**C**) pioglitazone versus LPS; (**D**) T0070907 versus LPS. The ∆PSI values of each ASE are presented on the X-axis, and the negative logarithmic adjusted *p*-value is presented on the Y-axis. Horizontal lines are equal to the negative logarithmic value of the adjusted *p*-value cut-off (0.05). Colored dots represent different types of significant DASEs (adjusted *p*-value < 0.05), and grey dots represent non-significant ASEs. The most interesting DASEs are labeled with gene symbols. The plots generated in maser bioconductor software.

An analysis of DEGs (FDR < 0.05%) revealed a total of 169 protein-coding genes in the LPS treatment group compared with the untreated group. A total of 115 overexpressed and 54 underexpressed DEGs were identified. The Gene Ontology (GO) annotation of biological processes (BP) contains 36 terms, whereas 14 ontology terms have been classified as molecular functions (MF) and 4 terms as cellular components (CC) (Fig. 4). Detailed results of DEG and GO analyses are presented in Tables S3 and S4 in the Supplemental Materials. The responses of endometrial slices to LPS treatment indicate that five genes (*CXCL9, CXCL10, CXCL11, CCL19* and *CXCL12*) are involved in chemokine activity. The above DEGs and 40 other genes (including *CCL5, CCL19, IL34, IDO1* and *CASP1*) encode proteins involved in immune system processes. Moreover, LPS treatment of endometrial tissues induced changes in the expression of genes regulating defense (28 DEGs) and inflammatory (14 DEGs) responses. The induction of endometrial toxicity was associated with the production of cytokines which trigger the transcriptional mechanism that regulates inflammation. In the group of genes encoding the response to cytokines, 12 DEGs were upregulated (*IRF7, ICAM1, ACKR1, IL34, ISG15, UBD, CXCL10, CXCL11, CASP1, CCR7, CCL5* and *CXCL12),* whereas five DEGs were downregulated (*TREM2, PDE2A, SPOCK2, APOA1* and *ENSSSCG00000006418*). Moreover, seven DEGs (*ENSSSCG00000006380, IL34, CXCL10, VEGFD, CCR7, CCL5* and *CXCL12*) were involved in the regulation of leukocyte chemotaxis, whereas 41 DEGs (including *TAP1, TREM2, PTGES, SFRP2, CASP1, APOA1, PDK3* and *ACOD1*) were engaged in stress responses. Eighteen DEGs were assigned to four significant signaling pathways (KEGG). In this group, *CCL22, CXCL9, CXCL10, CXCL11, ENSSSCG00000036445, CCL19, CCR7, CCL5* and *CXCL12* were assigned to the chemokine signaling pathway*.* In addition to DEGs, 24 DElncRNAs were also identified in endometrial tissues after LPS administration. The correlation analysis revealed that *ENSSSCG00000041052* (protein-coding gene) was the only DEG that was expressed similarly to three lncRNAs (underexpression, r^2^ > 0.9) after LPS treatment (Fig. 2A). Detailed results of the DElncRNA analysis are presented in Table S5 in the Supplemental Materials.

### The effect of PPARγ agonists on differential gene expression in LPS-treated endometrium

The influence of two PPARγ agonists on the gene expression profile of LPS-treated endometrium was determined in this part of the study. The incubation of endometrial tissues in the presence of PGJ_2_ or pioglitazone upregulated the expression of 181 and 476 genes, respectively, and downregulated the expression of 19 and 123 genes, respectively, relative to the LPS-treated group. In the GO analysis, DEGs were assigned to 57 BP terms, 4 MF terms and 1 CC term after PGJ_2_ treatment, and to 67 BP terms, 3 MF terms and 2 CC terms after pioglitazone treatment (Fig. 4). In the PGJ_2_-treated group, seven DEGs (*EGR1*, *IGF1*, *ZC3H12A*, *IL6*, *TNFAIP3*, *CCL3L1* and *GHSR*) were engaged in the production of interleukin-1 beta, and 27 genes (including *IDO1*, *SERPINE1*, *IL1A*, *RSAD2*, *ADAR*, *NFKBIA*, *TNFAIP3*, *MAP3K14* and *TRIM8* etc.) encoded defense responses. Most of these genes were upregulated. Moreover, eight upregulated genes (*CSF2*, *IL1β*, *NFKBIA*, *CXCL8*, *CXCL2*, *IL6*, *TNFAIP3* and *CSF3)* were implicated in the IL-17 signaling pathway (KEGG:04,657). Pioglitazone treatment of the LPS-inflamed endometrium induced changes in the expression of 11 genes (*BMP2*, *CSF2*, *CSF1*, *ENSSSCG00000024759*, *EDN1*, *TNF*, *IL25*, *IL6*, *CCL3L1*, *WNT5A* and *THPO*) that regulate cytokine activity. The identified DEGs were also engaged in vasculature development (34) and apoptotic processes (70). Seven upregulated genes (*PMAIP1, GADD45B, SERPINE1, APAF1, SESN3, GADD45G* and *ENSSSCG00000010448)* and three downregulated genes (*CCNG1*, *TP53I3* and *CDK1*) were implicated in the p53 signaling pathway (KEGG:04,115). Detailed results of DEG and GO analyses are presented Tables S3 and S4 in the Supplemental Materials, respectively.

The treatment of endometrial tissues with PPARγ agonists induced changes in the expression of 35 lncRNAs exposed to PGJ_2_ and 109 lncRNAs exposed to pioglitazone. After PGJ_2_ treatment, 22 noncoding transcripts were correlated with DEGs (Fig. 2B). In the pioglitazone-treated group, 69 DElncRNAs were bound by a strong positive correlation (r^2^ > 0.9) with DEG expression (Fig. 2C). Moreover, 18 common DElncRNAs were correlated with DEGs after exposure to both agonists. In one case, *ENSSSCG00000051243* (protein-coding gene) was negatively correlated (r^2^ < − 0.9) with lncRNA—*ENSSSCG00000050854* (Fig. 2B). Detailed results of the DElncRNA analysis are presented in Table S5 in the Supplemental Materials.

### The effect of the PPARγ antagonist on differential gene expression in LPS-treated endometrium

The effect of the PPARγ antagonist on the gene expression profiles of LPS-treated endometrium was determined in this part of the study. A total of 557 DEGs were identified (adjusted *p*-value < 0.05). Of those, 452 were upregulated and 105 were downregulated. In a functional cluster analysis, DEGs were annotated to 54 BP GO terms and six CC GO terms, as well as six signaling pathways in the KEGG database (Fig. 3). These DEGs were engaged in apoptosis regulation, RNA splicing, cell death and other processes. The GO analysis revealed that the genes involved in the regulation of apoptosis (including *RTKN2, CHST11, ZNF473, PTGIS* and *CD44*) and cell death (including *PIK3R1, CSF2, DOCK8, IGF1, GADD45G* and *DAPK1* etc.) were mostly overexpressed. In addition, 11 upregulated genes (*PIK3R1, CSF2, IL1β, ENSSSCG00000006286, MLKL, EDN1, CXCL2, IL6, TNFAIP3, MAP3K14* and *ENSSSCG00000010448)* were implicated in the TNF signaling pathway (KEGG:04,668). Detailed results of DEG and GO analyses are presented in Tables S3 and S4, respectively, in the Supplemental Materials. In endometrial tissues incubated in the presence of LPS and the PPAR*γ* antagonist, the expression of 89 lncRNAs increased and the expression of 16 lncRNAs decreased. In the group of DElncRNAs, 81 unique transcripts were positively correlated with 220 DEGs (Fig. 2D). Thirty-two DElncRNAs were specific to endometrial tissues exposed to the PPARγ antagonist and were not identified in other groups. Detailed results of the DElncRNA analysis are presented in Table S5 in the Supplemental Materials.

### The effect of LPS treatment on differential alternative splicing events in the endometrium

The reads were aligned to the target genome to evaluate differences in the percent spliced-in (PSI) value. A total of 130 differential alternative splicing events (DASEs), including 86 protein-coding genes, were identified. The DASEs were allocated to the following alternative splicing types: seven—alternative 3′ splice site (A3), eight—5′ splice site (A5SS), 56—alternative first exon (AFE), two—alternative last exon (ALE), five—mutually exclusive exon (MXE), 10 – retention intron (RI) and 42—skipping exon (SE). Differential alternative splicing events are presented in Table S6 in the Supplemental Materials. The GO analysis demonstrated that the Fc gamma R-mediated phagocytosis pathway was regulated by alternative splicing. Five DAS genes *(CFL2, CFL1, GAB2, LIMK1* and *AKT1*) were involved in this signaling pathway (KEGG:04,666). Moreover, the presence of the hypoxia-inducible factor (*HIF1α;* ΔPSI = 0.16; AF) and prostaglandin-endoperoxide synthase 2 (*PTGS2;* ΔPSI = 0.11; AF), which are engaged in the response to LPS, indicates that LPS treatment increased the inclusion level of AFEs in both genes. Two biomarkers (*PTP4A3*; ΔPSI = − 0.12; SE and *DUSP5*; ΔPSI = − 0.14; RI) of DNA damage revealed that the PSI value decreased in the LPS-treated group. Additionally, multiple AFEs in *PEPD* (ΔPSI = − 0.5: AF), *PDP2 (*ΔPSI = − 0.42: AF) and *BCAR3* (ΔPSI = 0.20; AF) genes were modulated by LPS. All of the above genes play a crucial role in the response to oxidative stress.

### The effect of PPARγ agonists on differential alternative splicing events in LPS-treated endometrium

**Figure 5:**
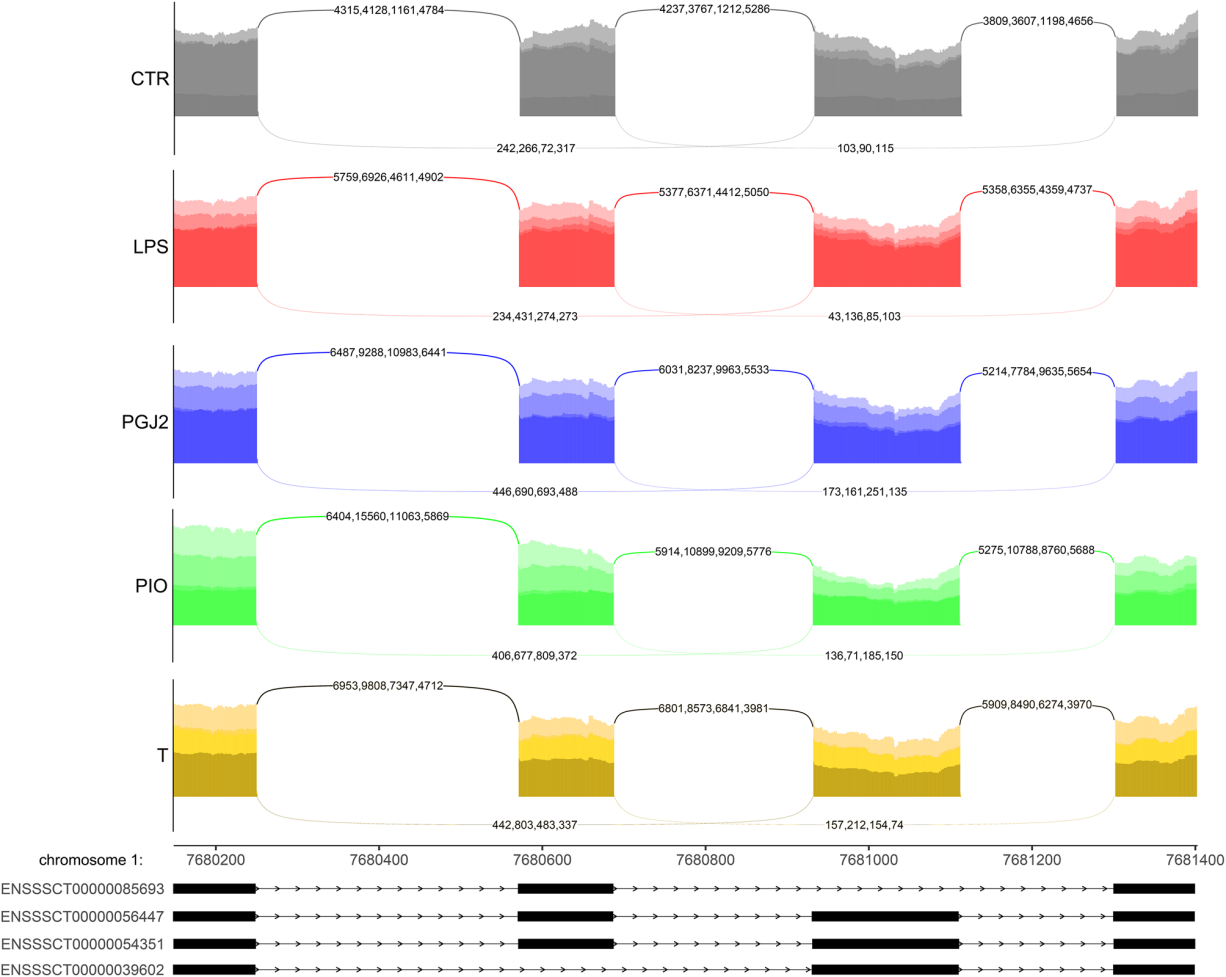
Sashimi plot (quantitative visualization) of differential alternative splicing events (DASEs) in the *SOD2* gene. Upper colored tracks represent the experimental conditions within mutually exclusive exons (MXEs). The numbers on curved lines indicate the number of counts for each splice junction. The middle track depicts the genomic coordinates of DASEs on the chromosome. The bottom tracks represent exons engaged in DASEs. The plot was generated in ggsashimi Python script.

A total of 172 and 211 DASEs were identified in endometrial tissues treated with PGJ_2_ and pioglitazone, respectively. The identified DASEs were allocated to the following splicing types: 10—A3, 8—A5, 46—AFE, 7—ALE, 6—MXE, 22—RI and 73—SE after PGJ_2_ treatment, and 19—A3, 8—A5, 58—AFE, 10—ALE, 8—MXE, 30—RI and 78—SE after pioglitazone treatment. Changes in PSI values were observed in 114 and 141 protein-coding genes after PGJ_2_ and pioglitazone treatment, respectively. The analysis demonstrated that PGJ_2_ participated in 96 splicing events and pioglitazone participated in 116 splicing events with a higher value of PSI (ΔPSI > 0.1). The GO and KEGG pathway enrichment analysis revealed that none of the DAS genes in the compared treatments were significantly enriched. Ninety-three DASEs were common for PGJ_2_ and pioglitazone treatments. However, a number of DAS genes play a crucial role in the regulation of oxidative stress. The study revealed changes in ASEs in *PRDX2* (ΔPSI = − 0.75; RI) and *SOD2* (ΔPSI = 0.24; MX) under exposure to pioglitazone (Fig. 5). *CLDND1* (ΔPSI = − 0.36; SE) and *PIGT* (ΔPSI = 0.42; SE) were identified only in endometrial tissues treated with PGJ_2_. The remaining oxidative stress biomarkers (*GJA1*, *ASS1*, *HIF1A*, *TTLL10*, *TATDN1*) changed alternative splice-sites after the administration of both agonists. Detailed results of the DAS analysis are presented in Table S6 in the Supplemental Materials.

### The effect of PPARγ antagonist on differential alternative splicing events in LPS-treated endometrium

A total of 200 DASEs were identified, including 13—A3, 12—A5, 50—AFE, 16—ALE, four—MXE, 35—RI and 70—SE. The antagonist treatment evoked 108 splicing events with a higher inclusion level (ΔPSI > 0.1) and 92 splicing events with a lower inclusion level (ΔPSI < 0.1). The greatest changes in the alternative splicing ratio were noted in *PPP1R21* (ΔPSI = 0.75; AL) and *GJB1* (ΔPSI = 0.59; AF), whereas the lowest inclusion levels were observed in *SEC24B* (ΔPSI = − 0.75; AF) and *ERI3* (ΔPSI = − 0.65; AL). A second DASE was observed in both genes: AFE (ΔPSI = -0.37) in *SEC24B* and ALE (ΔPSI = − 0.43) in *ERI3*. A functional clustering analysis revealed that in endometrial tissues exposed to the PPARγ antagonist, DAS genes were annotated to four CC GO terms associated with intercellular anatomical structures (92 DAS genes) and intercellular organelles (79 DAS genes). Differential alternative splicing events involved in the regulation of apoptosis, i.e., *BCL9* (ΔPSI = − 0.22, AS; ΔPSI = − 0.22, A3) and *PIK3R1* (ΔPSI = 0.02; SE), were also identified. It should also be noted that the PPARγ antagonist changed the ASEs of *PRDX2* (ΔPSI = − 0.5; RI) in the same manner as pioglitazone. After exposure to the PPARγ antagonist, the genes (*HSBP1L1—*ΔPSI = − 0.16, *MED25—*ΔPSI = -0.11 and *SEC24B—*ΔPSI = − 0.75, ΔPSI =− 0.37) annotated to GO terms associated with DNA damage and the response to oxidative stress were characterized by lower PSI values in the first and last exons. A somewhat higher potential for intron retention was noted only in *NQO2 *(-ΔPSI = 0.11, RI) Detailed results of the DAS analysis are presented in Table S6 in the Supplemental Materials.

### Real-time PCR validation

To validate the results of RNA-Seq, two DEGs (*CCL5, CCL19*) in the LPS-treated group and the untreated group, and four DEGs (*CDK1, CCNA1, CCNG1, TNFAIP3*) in the PPARγ-treated group and the LPS-treated group were selected for real-time PCR. The real-time PCR expression patterns of the tested DEGs were in agreement with RNA-Seq results (Fig. 1 in the Supplemental Materials).

## Discussion

Acute or chronic inflammations and bacterial infections in the female reproductive system contribute to infertility, endometrial polyps, miscarriage and diseases associated with abnormal placentation^1^. According to a growing number of researches, effective therapies are needed to modulate endometrial immune functions^23,24^. To address this problem, this study was undertaken to determine the potential role of PPARγ ligands in the regulation of inflammatory processes in the endometrium. To the best of our knowledge, this is the first study to comprehensively analyze the in vitro effect of PPARγ ligands on LPS-induced inflammation of the porcine endometrium during the follicular phase of the estrous cycle (high estrogen levels, low progesterone levels).

Interestingly, the gene expression profile in LPS-treated endometrium was different in the follicular phase (current study) and the mid-luteal phase of the estrous cycle^22^. In our previous study, 222, 3, 4, and 62 DEGs were identified after treatment with LPS, PGJ_2_, pioglitazone or T0070907, respectively. Significantly more genes were identified in the present study, which could be attributed to differences in sex steroid levels in various phases of the estrous cycle (high in the mid-luteal phase). Progesterone suppresses the innate immune function of the endometrium^25^.

In the present study, 169 DEGs were identified in the porcine endometrium after in vitro treatment with LPS, including 115 upregulated and 54 downregulated genes. The group of overexpressed genes included genes encoding proinflammatory chemokines *CCL5*, *CCL19*, *CCL22, CXCL9, CXCL10, CXCL11, CXCL12* and *CXCL13*. These chemotactic cytokines coordinate the positioning of cells, including immune system cells. Chemokines CXCL9, CXCL10, CXCL11, CXCL12, CXCL13 are regarded as inflammatory biomarkers in different tissues^26^. In turn, CCL5 induces the in vitro migration and recruitment of T cells, dendritic cells, eosinophils, NK cells, mast cells and basophils, whereas CCL19—expressed constitutively—regulates lymphocytes and dendritic cells^27^. Chemokine CCL22 induces the migration of CCR4+, Th2 and Treg cells^28^. All of the above chemokines have been found to be overexpressed upon LPS stimulation^28,29^. Our previous study revealed that the use of LPS in the proposed experimental model induced an inflammatory response in the porcine endometrium.

In the current study, PPARγ ligands influenced the transcriptome in the porcine endometrium after LPS-induced inflammation during the follicular phase of the estrous cycle. PPARγ agonists, PGJ_2_ or pioglitazone, increased the expression of 181 and 476 genes, but decreased the expression of 19 and 123 genes, respectively. After T0070907 treatment, 452 DEGs were upregulated and 105 DEGs were downregulated. Only the most interesting genes are discussed below.

The study revealed an interesting relationship between PPARγ ligands and the expression of genes involved in DNA damage response, such as *GADD45β, CDK1, CCNG1, CCNA1* and *ATM*. LPS stimulates the production of ROS, RNS and NO^5,30^ which contribute to DNA damage in different types of cells, including the endometrium^8^. Certain repair mechanisms are initiated in response to DNA damage, including the activation of the DNA damage checkpoint that arrests cell cycle progression^31^. *GADD45β* (growth-arrest and DNA-damage inducible 45 beta) seems to be a particularly interesting gene in the context of DNA damage response, regulation of inflammation and reproductive pathologies. GADD45β belongs to the GADD45 family whose members participate in the expression of genes regulating cell cycle checkpoint control, apoptosis, cell damage and other processes regulating cell growth. It has been reported that GADD45β plays an important role in G2/M arrest^32,33^ by inhibiting CDK1 activity and arresting the cell at the G2/M checkpoint^33,34^. Furthermore, Jiang et al. (2016) found a link between GADD45β and NF-E2-related factor 2 (NRF2), an antioxidant transcription factor that protects cells from ROS-induced damage by modulating the expression of cytoprotective and antioxidant genes. NRF2-GADD45β protects human embryonic kidney HEK293 cells against antimony-induced oxidative stress and apoptosis^35^. Additionally, GADD45β regulates various immunological processes because GADD45β-deficient dendritic cells produce less IFN-γ upon stimulation with LPS^36^. The recruitment of myeloid cells to the peritoneal cavity was also impaired after an LPS injection in mice lacking GADD45α or GADD45β^37^. The absence of GADD45β influences the in vitro differentiation of bone marrow cells into macrophage or granulocyte lineages^38^. It should also be noted that GADD45β can regulate the process of autophagy via the GADD45β-MEKK4-p38 signaling pathway^39,40^. *GADD45β* mRNA has been identified in human and rodent reproductive tissues^41,42^. Significant differences in *GADD45β* mRNA abundance were observed between women with chronic endometritis and healthy individuals^43^. Since endometriosis can be associated with a reduced level of autophagy, GADD45β can participate in the pathogenesis of endometriosis^44,45^. Autophagy is a DNA damage response that plays a crucial role in cell survival. This catabolic pathway is activated when cells are deficient in nutrients. Impaired and defective autophagy increases the susceptibility of cells to DNA damage and chromosomal instability^46,47^.

The results of this study indicate that pioglitazone upregulated *GADD45β* expression in LPS-treated porcine endometrium. To the best of our knowledge, this relationship has never been previously observed in reproductive tissues, however various PPARγ agonists have been reported to stimulate GADD45 gene or protein expression in human coronary artery smooth muscle cells (HCSMC)^48^ and cholangiocarcinoma cells^49^. The activation of PPARγ promoted apoptosis and growth arrest at the G2/M checkpoint. We hypothesize that pioglitazone, via *GADD45β,* could affect the DNA damage response during LPS-induced inflammation of the porcine endometrium*.* This influence could be mediated by CDK1 because pioglitazone inhibited *CDK1* expression. These findings elucidate the potential mechanisms by which PPARγ controls the cell cycle in an inflamed endometrium.

Cyclin-dependent kinases (CDKs) contain a specific catalytic core and cooperate with cyclins (regulatory subunits) that control kinase activity and substrate specificity. In the present study, PPARγ antagonist T0070907 inhibited the expression of cyclin A1 (*CCNA1*). CCNA1 levels are low in the G0 phase and increase in the early G1 phase^50^. CCNA1 is involved in G1 to S progression in somatic cells and plays an anti-apoptotic role in the DNA damage response^51,52^. Moreover, CCNA1 is strongly involved it the regulation of cancer progression and is consistently overexpressed in recurrent and chemoresistant tumors and cancer cell lines^53,54^. It has been reported that the expression of cyclin A1 was sufficient to enhance paclitaxel resistance, whereas the siRNA-induced decrease in cyclin A1 expression in the ovarian carcinoma cell line sensitized cells to paclitaxel cytotoxicity^54^. The present study demonstrated that *CCNA1* expression in LPS-stimulated porcine endometrium decreased when PPARγ was blocked by the antagonist. Although this study did not explore a tumor model, the presented results open up new avenues for research on cancer and various pathologies, as well as PPARγ activity and CCNA1.

ATM kinase is considered a master regulator of the cellular response to DNA double-strand breaks (DSBs), and it protects the integrity of the genome in mammalian cells by regulating the activation of cell cycle checkpoints^55^. For instance, ATM activation by ROS promotes endothelial proliferation by suppressing the accumulation of ROS through a feedback mechanism in a model of ischemic retinopathy^7^. Interestingly, PPARγ blocking with a specific antagonist in an LPS-stimulated endometrium upregulated the expression of *ATM*. ATM is activated in response to oxidative stress, and PPARγ blocking intensified this process^56^.

In the current study, the PPARγ agonist pioglitazone inhibited *CCNG1* expression in an LPS-stimulated porcine endometrium. Cyclin G1 has been found to act as oncogenic protein, and it was overexpressed in various types of uterine, ovarian, cervical and breast tumors^57^. Cyclin G is also involved in G2/M arrest in response to DNA damage, and it facilitates TNF-induced apoptosis^57^. To the best of our knowledge, only a single study reported on a significant decrease in cyclin G1 protein levels, without changes in the levels of the corresponding mRNA, in PPARγ −/− and PPARγ +/+ embryonic stem cells exposed to thiazolidinediones (troglitazone, ciglitazone)^58^. Therefore, the cited results are only partially consistent with our findings. However, this discrepancy could be attributed to differences in the analyzed tissues/PPARγ ligands or the duration of in vitro cultures. Surprisingly, in the current study, both PPARγ ligands (agonist and antagonist) exerted similar inhibitory effects on the abundance of cyclin G1 mRNA. Such phenomena were frequently noted by our team and other researchers^19,22^. Several explanations are possible, but the most likely one is that intracellular metabolic pathways are activated independently of PPARγ. It should also be noted that some molecules, such as PGC1α, are capable of ligand-independent binding and activating PPARγ^59^.

The current study provided new information on ASEs in the porcine endometrium treated with LPS and PPARγ ligands. A total of 130 DASEs were identified after LPS administration, whereas 172 and 211 DASEs were identified after PGJ_2_ or pioglitazone treatment, respectively. Moreover, 200 DASEs were identified after T0070907 treatment. The ASEs in genes encoding PRDX2 and SOD2 are particularly interesting. Both genes are involved in antioxidant reactions that are crucial for the proper course of many physiological processes^60^. An imbalance between ROS and anti-oxidative defense mechanisms induces intracellular oxidative stress and may contribute to disease and cancer progression. Enzymatic and non-enzymatic antioxidants make up a highly effective system that protects cells against ROS-induced oxidative stress^60^. This group of cell protectants includes peroxiredoxins (PRDXs) which regulate hydrogen peroxide levels and influence the signal transduction pathways induced by cytokines^61^. Mitochondrial superoxide dismutase (SOD2) belongs to the first line antioxidant defense system and catalyzes the conversion of the superoxide radical (O2· −) into hydrogen peroxide (H_2_O_2_). The expression of this antioxidant enzyme was higher in an ectopic than a normal endometrium^62^. There is evidence to suggest that ROS regulate various physiological processes, such as oocyte maturation, ovulation, follicular and luteal steroidogenesis, implantation, and early embryonic development^63^. Research has demonstrated that ROS-scavenging enzymes are involved in uterine physiology, but the role of ROS and antioxidant enzymes in endometrial functions has not been fully elucidated and future research is required^64^.

Long non-coding RNAs (lncRNAs) are non-coding genetic transcripts with a length of more than 200 nucleotides. Recent evidence indicates that lncRNAs participate in the regulation of various biological processes, such as tumor initiation, growth and metastasis, by initiating epigenetic, transcriptional and post-transcriptional mechanisms^65^. In this study, 25 lncRNAs were expressed after LPS treatment, whereas 109, 35 and 105 lncRNAs were expressed after treatment with pioglitazone, PGJ_2_ and T0070907, respectively. A comprehensive analysis of lncRNA profiles after pioglitazone treatment revealed the presence of *ENSSSCG00000047709* and *ENSSSCG00000042682* that positively regulated SMAD family member 7 (*SMAD7*). SMADs can act as transcription factors and central mediators in the canonical TGF-β signaling pathway^66^. SMAD7 is a potent modulator of the TGF-β family. SMAD7 has been found to inhibit TGF-β and BMP signaling through a negative feedback mechanism by blocking the activity of a type I receptor. SMAD7 expression is extensively regulated at the RNA level by noncoding RNAs (ncRNAs), such as microRNAs (miRNAs), circular RNAs (circRNAs) and long noncoding RNAs (lncRNAs)^66^. In addition to its inhibitory effect on TGFβ1 signaling, SMAD7 regulates the expression and function of several molecules involved in inflammation control. Moreover, it has been reported that SMAD7 protects against acute kidney injury by rescuing the G1 cell cycle arrest of tubular epithelial cells^67^.

## Conclusions

The in vitro effect of PPARγ ligands on the global transcriptome profile of an inflamed porcine endometrium during the follicular phase of the estrous cycle was described comprehensively in this study. The study demonstrated that PPARγ is involved in various immunological processes, including IL-1β production, IL-17 signaling pathway and defense response. Most of the described DEGs have been assigned to the p53 signaling pathway. The observation that pioglitazone regulates the expression of genes encoding the DNA damage response to stress conditions makes a valuable contribution to the existing body of knowledge. These findings open up new avenues for research on PPARγ mechanisms that control reproductive functions during inflammation.

## Materials and Methods

### Animals

The study was conducted on crossbred pigs (Large White × Polish Landrace) on days 18–20 of the estrous cycle (follicular phase; n = 4). The animals were aged 7–8 months, weighed approximately 100 kg, and were reared in a private farm. After slaughter, uterine horns were dissected, and the collected tissues were transported on ice in phosphate‐buffered saline (PBS) with antibiotics: 100 IU/mL of penicillin and 100 mg/mL of streptomycin (Polfa Tarchomin, Warsaw, Poland). The experimental material was collected in accordance with the national guidelines for animal care as well as ARRIVE guidelines. All procedures were approved by the Animal Ethics Committee of the University of Warmia and Mazury in Olsztyn, Poland.

### In vitro experiment

The procedure for collecting and incubating porcine endometrial tissue has been described previously^68^. In the laboratory, the endometrium was separated from the myometrium, washed with sterile PBS containing antibiotics, and placed on ice in a sterile Petri dish. Tissue slices (100 ± 10 mg, collected in one piece in duplicate from each animal) were incubated in the M199 medium (Sigma‐Aldrich, St. Louis, MO, USA) supplemented with 0.1% BSA fraction V (Roth, Germany) and antibiotics: nystatin (120 IU/mL, Sigma‐Aldrich) and gentamicin (40 mg/mL; Sigma‐Aldrich). The explants were pre‐incubated for two hours on a rocking platform in a water bath at 37 °C in an atmosphere of 95% O_2_ and 5% CO_2._ After incubation, the explants were treated with LPS (100 ng/ml, from *Escherichia coli*) for 24 h. Explants not treated with LPS were the control. The medium was removed, and the explants were incubated in an LPS-free medium for 6 h with PPARγ ligands: 15‐deoxy‐Δ12,14‐prostaglandin J_2_ (PGJ_2_; natural agonist; 10 μmol/L, Enzo Life Sciences Int., New York, NY, USA), pioglitazone (PIO; synthetic agonist; 1 μmol/L, Cayman Chemical Company, Ann Arbor, MI, USA), and T0070907 (T; antagonist; 1 μmol/L, Cayman Chemical Company). The control additionally contained dimethyl sulfoxide (DMSO, solvent for the tested PPAR ligands, total volume of 20 µl). The doses of the tested factors were selected based on the results of our preliminary study and literature data^69,70^. After incubation, tissue explants were washed with PBS and frozen at − 80 °C for total RNA isolation. Tissue slices were stored until RNA-Seq and real-time PCR analysis.

### RNA isolation, library preparation and sequencing procedure

Total RNA from 20 samples (4 pigs × 5 treatments) was isolated with the RNeasy Mini Kit (Qiagen, Germany) according to the manufacturer’s protocol. The purity and concentration of the isolated RNA was measured with the Tecan Infinite M200 plate reader (Tecan Group Ltd., Switzerland). Sample degradation was evaluated in the Agilent Bioanalyzer 2100 (Agilent Technology, USA). Twenty RNA samples with an RNA Integrity Number (RIN) > 7 were selected for future analysis. The library preparation and sequencing procedure was described previously^22^. In brief, the library was prepared with the TruSeq Stranded mRNA LT Sample Prep Kit (Illumina, San Diego, CA, USA). Genetic material was fragmented, and RNA was transcribed into cDNA using reverse transcriptase. Fragments of double-stranded cDNA were marked with specific adapters for each library. The generated cDNA fragments were strand-specific. Finally, pooled libraries were sequenced on the Illumina NovaSeq 6000 platform with 2 × 150 bp paired-end (PE) chemistry.

### Quality controls and genome mapping

The quality of raw paired-end reads was controlled with FastQC and Trimmomatic, and sequences were processed to (a) minimum length > 120 bp, (b) PHRED score > 20, (c) cropped to equal length. High-quality trimmed reads were aligned to the Sus_scrofa 11.1 genome assembly with reference to the ENSEMBL annotation (release 98) using the Spliced Transcripts Alignment to a Reference (STAR) aligner. Mapping results were indexed and sorted by coordinates. Gene expression values (read counts) were reconstructed by compiling ballgown files and the prepDE.py script. Sequencing data (PRJEB45635) were submitted to the European Nucleotide Archive (ENA).

### Differentially expressed genes

An analysis of DEGs and the corresponding false discovery rate (FDR < 0.05) was performed using DESeq2. Changes in the gene expression pattern in the porcine endometrium on days 18–20 of the estrous cycle after in vitro treatment with LPS and/or PPARγ ligands (agonists: 15d-prostaglandin J_2_ (PGJ_2_) or pioglitazone (PIO); and antagonist T0070907 (T)) were determined by high-throughput transcriptome sequencing. The transcriptomic effects of treatment with LPS and PPARγ ligands were examined in four comparisons: LPS versus control, PGJ_2_ versus. LPS, PIO versus LPS, and T versus LPS. Additionally, fragments per kilobase of transcript per million mapped reads (FPKM) were calculated as a normalized expression measure that depends on sequencing depth and the length of genomic features. The enrichment of the main biological processes and the metabolic pathways in DEGs were identified by enrichGO and enrich KEGG methods implemented in the ontology-based clusterProfiler R package^71^. In the functional enrichment analysis, the cut-off criteria were: organism, pig; ont, CC, MF or BP; P-adjust value cut-off, 0.05; P-adjust method, BH. To draw expression and functional profiles, the ggplot2, circlize, GOplot R bioconductor packages were used.

### lncRNA identification and analysis

A multi-step pipeline was used to identify lncRNA candidates in the porcine endometrium, as previously described by Paukszto et al. (2019)^72^. In brief, transcripts with protein-coding Ensembl class code annotation and low-expressed transcripts were removed. Transcripts with a single exon and a sequence length shorter than 200 bp were then filtered out. Coding potential was estimated with the Coding Potential Calculator (CPC2), PLEK and FlExible Extraction of LncRNAs (FEELnc)^45,73^. The set of predicted lncRNA transcripts and annotated lncRNAs (with ENSEMBL gene IDs) constituted the final pool of endometrial lncRNAs. The obtained lncRNAs were compiled with DEGs, and common transcripts were identified as differentially expressed lncRNAs (DElncRNAs). Pairs of coregulated lncRNAs and DEGs were identified based on the values of the correlation coefficient and Euclidean distance (absolute value of r^2^ > 0.9). Next, the expression of each lncRNA identified in the treated endometrium was compared with the expression of the respective lncRNA identified in the control samples. This approach enabled the identification of differentially expressed lncRNAs (DElncRNAs); *p*-adjusted < 0.05 and log_2_ fold change (log_2_FC) ≥ 1.0; DESeq2 software). The identified DELs and DEGs were also compared based on the values of the correlation coefficient and Euclidean distance.

### Differential alternative splicing events

Differences in alternative splicing were predicted by a super-fast pipeline for alternative splicing analysis (SUPPA v.2)^71^. Trimmed paired-end reads of equal length (90 bp) were used to calculate the percent spliced-in (PSI) value for all ASEs. Reads were remapped to the reference transcriptome using Salmon software. Differential alternative splicing events for each of the four comparisons (LPS vs. control, PGJ_2_ vs*.* LPS, PIO vs. LPS and T vs. LPS) were statistically tested (FDR < 0.05). Splicing events with ΔPSI > 0.1 were classified as significant. Alternative events were classified into seven types in SUPPA software: alternative 5′ splice site (A5SS), alternative 3′ splice site (A3SS), mutually exclusive exons (MXE), retention intron (RI), skipping exon (SE), alternative first exon (AFE) and alternative last exon (ALE). Alternative splicing events were visualized in the maser package and ggsashimi python scripts.

### Real-time PCR validation

Differentially expressed genes were validated by real-time PCR with the use of the AriaMx real-time PCR System (Agilent Technology, USA), as described previously^22^. Primer sequences (Supplemental Table S6) for reference and target genes (*CCL5, CCL19, CDK1, CCNA1, CCNG1, TNFAIP3*) were designed using Primer Express Software 3 (Applied Biosystems, USA). PCR reaction mixtures with a final volume of 25 μl consisted of cDNA (4 ng), 300 μM of each primer, 12.5 μl of the Power SYBR Green PCR Master Mix (Applied Biosystems, USA), and RNase-free water. The abundance of the tested mRNAs was calculated using standard curves which were prepared by a serial dilution of a known amount of total RNA. Constitutively expressed *ACTB* and *GAPDH* genes were adopted as the reference genes, and the geometric means of expression levels were applied in the analysis. The results of real-time PCR were processed statistically in the Statistica software (Statsoft Inc. Tulsa, USA) with Student’s t-test and were expressed as means ± SEM. The results were regarded as statistically significant at *p* < 0.05.

## Supplementary Information

Supplementary Information 1.

Supplementary Information 2.

Supplementary Information 3.

Supplementary Information 4.

Supplementary Information 5.

Supplementary Information 6.

Supplementary Information 7.

## References

[1] Drizi A, Djokovic D, Laganà AS, van Herendael B (2020). Impaired inflammatory state of the endometrium: A multifaceted approach to endometrial inflammation. Current insights and future directions. Menopausal Rev..

[2] Chen L (2018). Inflammatory responses and inflammation-associated diseases in organs. Oncotarget.

[3] Burrell R (1994). Human responses to bacterial endotoxin. Circ. Shock.

[4] Deb K, Chatturvedi MM, Jaiswal YK (2004). Gram-negative bacterial endotoxin-induced infertility: A birds eye view. Gynecol. Obstet. Invest..

[5] Kim J-C (2012). Anti-inflammatory mechanism of PPARγ on LPS-induced pulp cells: Role of the ROS removal activity. Arch. Oral Biol..

[6] Pálmai-Pallag T, Bachrati CZ (2014). Inflammation-induced DNA damage and damage-induced inflammation: A vicious cycle. Microb. Infect..

[7] Yan S, Sorrell M, Berman Z (2014). Functional interplay between ATM/ATR-mediated DNA damage response and DNA repair pathways in oxidative stress. Cell. Mol. Life Sci..

[8] Jaiswal YK, Jaiswal MK, Agrawal V, Chaturvedi MM (2009). Bacterial endotoxin (LPS)–induced DNA damage in preimplanting embryonic and uterine cells inhibits implantation. Fertil. Steril..

[9] Földi J (2006). Bacterial complications of postpartum uterine involution in cattle. Anim. Reprod. Sci..

[10] Augoulea A, Alexandrou A, Creatsa M, Vrachnis N, Lambrinoudaki I (2012). Pathogenesis of endometriosis: The role of genetics, inflammation and oxidative stress. Arch. Gynecol. Obstet..

[11] Khan KN (2018). Bacterial contamination hypothesis: a new concept in endometriosis. Reprod. Med. Biol..

[12] Bane K (2021). Endometrial DNA damage response is modulated in endometriosis. Hum. Reprod..

[13] Grassi T (2015). Mismatch repair system in endometriotic tissue and eutopic endometrium of unaffected women. Int. J. Clin. Exp. Pathol..

[14] Bogacka I, Kurzynska A, Bogacki M, Chojnowska K (2015). Peroxisome proliferator-activated receptors in the regulation of female reproductive functions. Folia Histochem. Cytobiol..

[15] Takada I, Makishima M (2020). Peroxisome proliferator-activated receptor agonists and antagonists: A patent review (2014-present). Expert Opin. Ther. Pat..

[16] Barak Y (1999). PPARγ is required for placental, cardiac, and adipose tissue development. Mol. Cell.

[17] Vitti M (2016). Peroxisome proliferator-activated receptors in female reproduction and fertility. PPAR Res..

[18] Parast MM (2009). PPARγ Regulates Trophoblast Proliferation and Promotes Labyrinthine Trilineage Differentiation. PLoS One.

[19] Kunicka Z, Kurzynska A, Szydlowska A, Mierzejewski K, Bogacka I (2019). Peroxisome proliferator-activated receptor gamma ligands affect NF-kB and cytokine synthesis in the porcine endometrium-An in vitro study. Am. J. Reprod. Immunol..

[20] Korbecki J, Bobiński R, Dutka M (2019). Self-regulation of the inflammatory response by peroxisome proliferator-activated receptors. Inflamm. Res..

[21] Song EA, Lim JW, Kim H (2017). Docosahexaenoic acid inhibits IL-6 expression via PPARγ-mediated expression of catalase in cerulein-stimulated pancreatic acinar cells. Int. J. Biochem. Cell Biol..

[22] Mierzejewski K (2021). Transcriptome analysis of porcine endometrium after LPS-induced inflammation: Effects of the PPAR-gamma ligands in vitro. Biol. Reprod..

[23] Maybin JA, Critchley HOD, Jabbour HN (2011). Inflammatory pathways in endometrial disorders. Mol. Cell. Endocrinol..

[24] Robertson SA (2016). Corticosteroid therapy in assisted reproduction: Immune suppression is a faulty premise. Hum. Reprod..

[25] Cui L (2020). Progesterone inhibits inflammatory response in E. coli-or LPS-Stimulated bovine endometrial epithelial cells by NF-κB and MAPK pathways. Dev. Comp. Immunol..

[26] Dotan I (2010). CXCL12 Is a constitutive and inflammatory chemokine in the intestinal immune system. Inflamm. Bowel Dis..

[27] Muller G, Hopken UE, Lipp M (2003). The impact of CCR7 and CXCR5 on lymphoid organ development and systemic immunity. Immunol. Rev..

[28] Iellem A (2000). Inhibition by IL-12 and IFN-α of I-309 and macrophage-derived chemokine production upon TCR triggering of human Th1 cells. Eur. J. Immunol..

[29] Pickens SR (2011). Characterization of CCL19 and CCL21 in rheumatoid arthritis. Arthritis Rheum..

[30] Nathan C, Shiloh MU (2000). Reactive oxygen and nitrogen intermediates in the relationship between mammalian hosts and microbial pathogens. Proc. Natl. Acad. Sci..

[31] Sancar A, Lindsey-Boltz LA, Ünsal-Kaçmaz K, Linn S (2004). Molecular mechanisms of mammalian DNA repair and the DNA damage checkpoints. Annu. Rev. Biochem..

[32] Smith M (1994). Interaction of the p53-regulated protein Gadd45 with proliferating cell nuclear antigen. Science.

[33] Hollander MC, Fornace AJ (2002). Genomic instability, centrosome amplification, cell cycle checkpoints and Gadd45a. Oncogene.

[34] Zhan Q (1999). Association with Cdc2 and inhibition of Cdc2/Cyclin B1 kinase activity by the p53-regulated protein Gadd45. Oncogene.

[35] Jiang X (2016). The protective role of Nrf2-Gadd45b against antimony-induced oxidative stress and apoptosis in HEK293 cells. Toxicol. Lett..

[36] Lu B, Ferrandino AF, Flavell RA (2004). Gadd45β is important for perpetuating cognate and inflammatory signals in T cells. Nat. Immunol..

[37] Salerno DM, Tront JS, Hoffman B, Liebermann DA (2012). Gadd45a and Gadd45b modulate innate immune functions of granulocytes and macrophages by differential regulation of p38 and JNK signaling. J. Cell. Physiol..

[38] Gupta SK, Gupta M, Hoffman B, Liebermann DA (2006). Hematopoietic cells from gadd45a-deficient and gadd45b-deficient mice exhibit impaired stress responses to acute stimulation with cytokines, myeloablation and inflammation. Oncogene.

[39] Keil E (2013). Phosphorylation of Atg5 by the Gadd45β–MEKK4-p38 pathway inhibits autophagy. Cell Death Differ..

[40] Schmitz I (2013). Gadd45 proteins in immunity. Adv. Exp. Med. Biol..

[41] Groothuis PG, Dassen HHNM, Romano A, Punyadeera C (2007). Estrogen and the endometrium: Lessons learned from gene expression profiling in rodents and human. Hum. Reprod. Update.

[42] Yan Z (2019). Endometrial mesenchymal stem cells isolated from menstrual blood repaired epirubicin-induced damage to human ovarian granulosa cells by inhibiting the expression of Gadd45b in cell cycle pathway. Stem Cell Res. Ther..

[43] Di Pietro C (2013). Altered transcriptional regulation of cytokines, growth factors, and apoptotic proteins in the endometrium of infertile women with chronic endometritis. Am. J. Reprod. Immunol..

[44] Mei J (2015). Estrogen promotes the survival of human secretory phase endometrial stromal cells via CXCL12/CXCR4 up-regulation-mediated autophagy inhibition. Hum. Reprod..

[45] Kang Y-J (2017). CPC2: a fast and accurate coding potential calculator based on sequence intrinsic features. Nucl. Acids Res..

[46] Vessoni AT, Filippi-Chiela EC, Menck CF, Lenz G (2013). Autophagy and genomic integrity. Cell Death Differ..

[47] Anand SK, Sharma A, Singh N, Kakkar P (2020). Entrenching role of cell cycle checkpoints and autophagy for maintenance of genomic integrity. DNA Repair (Amst).

[48] Bruemmer, D. *et al.* Regulation of the growth arrest and DNA damage-inducible gene 45 (GADD45) by peroxisome proliferator-activated receptor γ in vascular smooth muscle cells. *Circ. Res.* **93**, (2003). 10.1161/01.RES.0000088344.15288.E6 12881480

[49] Han C (2003). PPARγ ligands inhibit cholangiocarcinoma cell growth through p53-dependent GADD45 and p21WAF1/Cip1 pathway. Hepatology.

[50] Yang R, Morosetti R, Koeffler HP (1997). Characterization of a second human cyclin A that is highly expressed in testis and in several leukemic cell lines. Cancer Res..

[51] Ji P (2005). Cyclin A1, the alternative A-type cyclin, contributes to G1/S cell cycle progression in somatic cells. Oncogene.

[52] Woo SH (2014). Implications of caspase-dependent proteolytic cleavage of cyclin A1 in DNA damage-induced cell death. Biochem. Biophys. Res. Commun..

[53] Wegiel B (2005). A role for cyclin A1 in mediating the autocrine expression of vascular endothelial growth factor in prostate cancer. Oncogene.

[54] Huang K-C (2016). Cyclin A1 expression and paclitaxel resistance in human ovarian cancer cells. Eur. J. Cancer.

[55] Guo Z, Deshpande R, Paull TT (2010). ATM activation in the presence of oxidative stress. Cell Cycle.

[56] Aleshin S, Reiser G (2013). Role of the peroxisome proliferator-activated receptors (PPAR)-α, β/δ and γ triad in regulation of reactive oxygen species signaling in brain. Biol. Chem..

[57] Seo HR (2006). Cyclin G1 overcomes radiation-induced G2 arrest and increases cell death through transcriptional activation of cyclin B1. Cell Death Differ..

[58] Palakurthi SS, Aktas H, Grubissich LM, Mortensen RM, Halperin JA (2001). Anticancer effects of thiazolidinediones are independent of peroxisome proliferator-activated receptor gamma and mediated by inhibition of translation initiation. Cancer Res..

[59] Wu Y, Chin WW, Wang Y, Burris TP (2003). Ligand and coactivator identity determines the requirement of the charge clamp for coactivation of the peroxisome proliferator-activated receptor γ. J. Biol. Chem..

[60] Birben E, Sahiner UM, Sackesen C, Erzurum S, Kalayci O (2012). Oxidative stress and antioxidant defense. World Allergy Organ. J..

[61] Wood ZA (2003). Peroxiredoxin evolution and the regulation of hydrogen peroxide signaling. Science.

[62] Chen C (2019). Mitochondria and oxidative stress in ovarian endometriosis. Free Radic. Biol. Med..

[63] Sugino N (2005). Reactive oxygen species in ovarian physiology. Reprod. Med. Biol..

[64] Al-Gubory KH, Bolifraud P, Garrel C (2008). Regulation of key antioxidant enzymatic systems in the sheep endometrium by ovarian steroids. Endocrinology.

[65] Chen X, Yan CC, Zhang X, You Z-H (2016). Long non-coding RNAs and complex diseases: from experimental results to computational models. Brief. Bioinform.

[66] de Ceuninck van Capelle, C., Spit, M. & ten Dijke, P. Current perspectives on inhibitory SMAD7 in health and disease. *Crit. Rev. Biochem. Mol. Biol.* **55**, 691–715 (2020). 10.1080/10409238.2020.1828260 33081543

[67] Fu S (2017). Smad7 protects against acute kidney injury by rescuing tubular epithelial cells from the G1 cell cycle arrest. Clin. Sci..

[68] Kurzyńska A, Bogacki M, Chojnowska K, Bogacka I (2016). Peroxisome proliferator activated receptor ligands affect porcine endometrial steroids production during the estrous cycle and early pregnancy: an in vitro study. Czech J. Anim. Sci..

[69] Swangchan-Uthai T, Lavender CRM, Cheng Z, Fouladi-Nashta AA, Wathes DC (2012). Time course of defense mechanisms in bovine endometrium in response to lipopolysaccharide. Biol. Reprod..

[70] Czarzasta J, Andronowska A, Jana B (2014). Pro-and anti-inflammatory mediators change leukotriene B4 and leukotriene C4 synthesis and secretion in an inflamed porcine endometrium. Domest. Anim. Endocrinol..

[71] Trincado JL (2018). SUPPA2: fast, accurate, and uncertainty-aware differential splicing analysis across multiple conditions. Genome Biol..

[72] Paukszto L (2020). Transcriptome, spliceosome and editome expression patterns of the porcine endometrium in response to a single subclinical dose of salmonella enteritidis lipopolysaccharide. Int. J. Mol. Sci..

[73] Wucher V (2017). FEELnc: A tool for long non-coding RNA annotation and its application to the dog transcriptome. Nucl. Acids Res.

