# Supplementary material for: PPARγ regulates the expression of genes involved in the DNA damage response in an inflamed endometrium
Source: Sci Rep. 2022 Mar 7;12:4026. doi: 10.1038/s41598-022-07986-8 (PMC8901773; doi:10.1038/s41598-022-07986-8)
Supplement: Supplementary file 7 — Supplementary Information 7. [file 41598_2022_7986_MOESM7_ESM.pdf]

Supplementary information to

PPARγ regulates the expression of genes involved in DNA damage repair in inflamed endometrium – transcriptome analysis

Karol Mierzejewski[1], Łukasz Paukszto[2], Aleksandra Kurzyńska[1], Zuzanna Kunicka[1], Jan P Jastrzębski[2], Karol G Makowczenko[1], Monika Golubska[1], Iwona Bogacka[1,*]

[1] University of Warmia and Mazury in Olsztyn, Faculty of Biology and Biotechnology, Department of Animal Anatomy and Physiology; Oczapowskiego 1a, 10-719 Olsztyn, Poland

[2] University of Warmia and Mazury in Olsztyn, Faculty of Biology and Biotechnology, Department of Plant Physiology, Genetics and Biotechnology; Oczapowskiego 1a, 10-719 Olsztyn, Poland

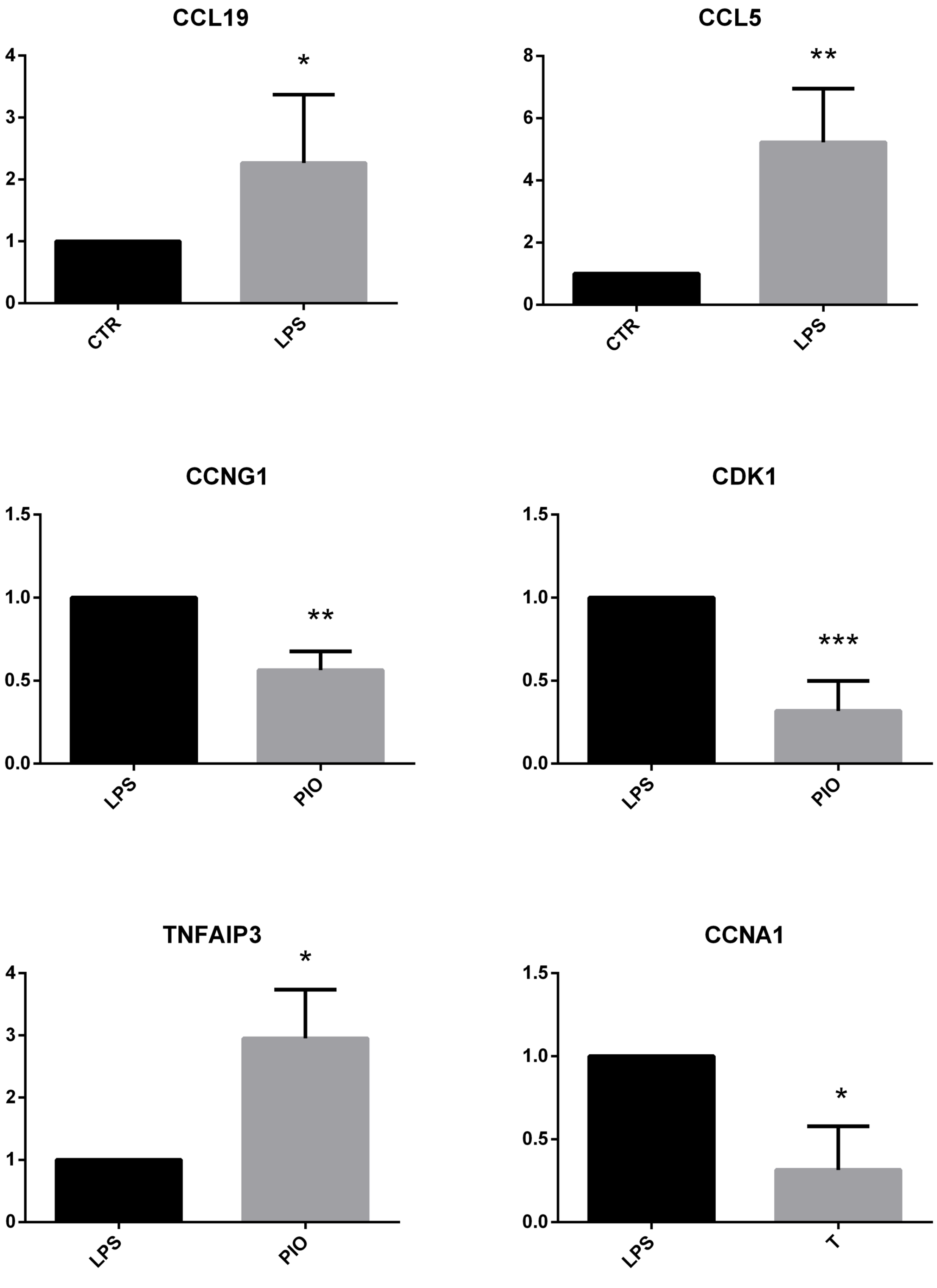

Supplementary Figure 1

Real-time PCR validation of RNA-seq results for random DEGs. Validation was performed for *CCL5*, *CCL19*, *CDK1*, *CCNA1*, *CCNG1* and *TNFAIP3*.

Gene	Forward	Reverse	bp
CCL5	ACACCACACCCTGCTGTTTT	TCTTCTCTGGGTTGGCACAC	150
CCL19	TTCGAGCCTTTCGCTACCTG	AGTCTCTTGATGATGCGCTCC	130
CDK1	TGCTTATGCAAGACTCCAGGT	TCCCTTGCAGGATTTGGTACA	141
CCNA1	GCTGAAATGAGATACCGGCCT	AGTCCACCAGAATCGTTCGC	85
CCNG1	TGGATTGTTTCTGGGCGTACT	TCAGGATTGTGGAGAAAGGCT	147
GAPDH	ACATCATCCCTGCTTCTACCG	CCAGTGAGCTTCCCGTTGAG	76
TNFAIP3	GTGACCCTGAAGGACAGTGG	CGAATCTTCCTCGCTCTCTGT	70
ACTB	ACATCAAGGAGAAGCTCTGCTACG	GAGGGGCGATGATCTTGATCTTCA	366

Supplementary Table S 7

Primers used in Real-time PCR validation of DEGs.

Supplementary materials

Supplementary Table S 1

Summary of the sequencing depth and mapping reads according to the porcine reference genome.

Supplementary Table S 2

The paired-end bases mapped to the coding, UTR, intronic and intergenic regions in ENSEMBL pig genome.

Supplementary Table S 3

Differentially expressed genes identified in the porcine endometrium after LPS and PPARγ ligands treatment.

Supplementary Table S 4

Results of Gene Ontology enrichment analysis of DEGs significantly modulated by subclinical LPS and PPARγ ligands in porcine endometrium.

Supplementary Table S 5

Differentially long non-coding RNAs induced by LPS and PPARγ ligands treatment.

Supplementary Table S 6

Differentially alternative splicing events after LPS and PPARγ ligands treatment.
